# Longitudinal Variation of Amino Acid Levels in Human Milk and Their Associations with Infant Gender

**DOI:** 10.3390/nu10091233

**Published:** 2018-09-05

**Authors:** Joris H. J. van Sadelhoff, Bert J. M. van de Heijning, Bernd Stahl, Sonia Amodio, Edmond H. H. M. Rings, M. Luisa Mearin, Johan Garssen, Anita Hartog

**Affiliations:** 1 Danone Nutricia Research, Early Life Nutrition, Uppsalalaan 12, 3584 CT Utrecht, The Netherlands; j.h.j.vansadelhoff@uu.nl (J.H.J.v.S.); bert.vandeheijning@danone.com (B.J.M.v.d.H.); Bernd.STAHL@danone.com (B.S.); Sonia.AMODIO@nutricia.com (S.A.); Johan.Garssen@danone.com (J.G.); 2 Division of Pharmacology, Utrecht Institute for Pharmaceutical Science, Faculty of Sciences, Utrecht University, Universiteitsweg 99, 3584 CG Utrecht, The Netherlands; 3 Department of Pediatrics, Erasmus Medical Center, Doctor Molewaterplein 40, 3015 GD Rotterdam, The Netherlands; e.rings@erasmusmc.nl; 4 Department of Pediatrics, Leiden University Medical Center, Albinusdreef 2, 2333 ZA Leiden, The Netherlands; M.L.Mearin_Manrique@lumc.nl

**Keywords:** breastfeeding, free amino acids, glutamine, glutamate

## Abstract

It is discussed that specific amino acids (AAs) have functional roles in early life. Understanding the AA composition in human milk (HM) during lactation assists in specifying these roles. To this end we assessed the levels of free AAs (FAAs), total AAs (free and bound, TAAs) and protein levels in HM in the first 6 months of lactation, and evaluated possible associations with infant gender. HM samples of 25 healthy Dutch mothers participating in the PreventCD study were collected monthly during the first 6 months of lactation. Of the participating mothers, 12 gave birth to a boy and 13 gave birth to a girl. Analyses of the HM samples revealed that levels of free glutamate, glutamine, aspartate, glycine, and serine significantly increased during months 1–3 of lactation, both in absolute sense and relative to TAA levels. Evaluation of gender differences by mixed model analyses revealed an association between female infant gender and higher protein content (*p* = 0.0465) and TAA content (*p* = 0.0362) in HM during the first 3 months of lactation. Furthermore, there was a tendency for an association of male infant gender with higher levels of free glutamine (*p* = 0.0948) in HM during the first 3 months of lactation. These results show that FAA, TAA and protein levels in HM display a time-specific occurrence during lactation. Moreover, although confirmation is necessary in view of the small sample size, this study indicates that the AA composition in HM shows differential effects of the infant’s sex.

## 1. Introduction

Human milk (HM) is the best source of infant nutrition providing the energy, nutrients, and non-nutritive factors for an optimal growth and development. Breastfeeding is shown to have beneficial effects on the infant beyond nutrition. It is known to support gastrointestinal development by stimulating intestinal bacterial colonization and barrier formation, and it provides immediate protection against infectious diseases [1,2]. Recent studies show that breastfeeding reduces mortality rates among infants and decreases the risk for developing certain types of allergies, autoimmune diseases and (late) metabolic diseases, protecting against type 2 and potentially also type 1 diabetes, as well as obesity [3,4,5]. The significant effects of breastfeeding on infant health and development urge the need to define and understand the composition and functionality of HM.

Proteins make up a significant part of the macronutrient composition of HM and contribute to its unique activity. Most of the proteins in HM are digested to provide the infant with an adequate supply of amino acids (AAs), which is critical for a normal growth and development [6]. Although AAs are released from milk proteins, HM also contains free (i.e., not part of protein) AAs (FAAs), for reasons not well understood [7].

Multiple studies investigated the dynamics of FAA composition in HM throughout lactation. As summarized by the systematic review of Zhang et al., levels of most FAAs remain quite stable within the first months of lactation, whereas free glutamine and glutamate, among others, increase [7]. As infant milk volume intake per kg body weight elevates with progressing lactation, the relative intake of free glutamine and glutamate of the infant increases, suggesting specific roles for these FAAs in early life. It has been shown that free glutamine is an energy substrate for intestinal cells and is important for providing ketoglutaric acid for the citric acid cycle [8], whereas free glutamate is indicated to confer beneficial physiological effects in the developing oral cavity and gut, e.g., regulating food intake and satiety and modulating gut and immune-development [9,10].

The role of specific components in HM in improving disease outcome is still unclear. It is speculated that the dynamics of specific components in HM, including oligosaccharides and fatty acids, influence immune maturation and (allergic) disease development [11]. Studies have shown gender differences in the occurrence allergic diseases, i.e., the prevalence of asthma, wheeze and potentially food allergy is more frequent among male infants, whereas coeliac disease (CD) occurs more frequently in female infants [12,13,14,15]. In compliance with these findings it is argued that HM composition might be dependent on the infant’s sex, endorsed by studies indicating associations of infant gender with fat and energy content in HM [16,17]. Whether the composition and levels of AAs in HM during lactation is dependent on the infant’s gender has never been studied. Potential gender effects on AA composition in HM might provide new insights in how HM compositions contributes to early life development.

The objective of this study was to assess the changes in HM AA composition during lactation and to study the effect of gender, in order to contribute to the discussion of their role in the developing infant. Therefore, we measured FAA, TAA, and protein levels in HM sample series of 25 Dutch women during the first 6 months of lactation, and investigated whether AA and protein levels in HM differ between genders.

## 2. Materials and Methods 

### 2.1. Participants and Study Design

During 2007–2010, HM samples were collected from women participating in the PreventCD study (Current Controlled Trials number: ISRCTN74582487), a dietary-intervention study which has been described in detail elsewhere [15]. Briefly, term infants from 0 to 3 months of age and genotypically at risk of developing CD (i.e., the presence of DQ2 and DQ8 haplotypes) and with at least one family member with CD were recruited. Participating infants were randomly assigned to receive either 200 mg of wheat (intervention) or 200 mg of lactose (placebo) daily for 8 weeks starting at the age of 16 weeks. The primary outcome was the frequency of CD at 3 years of age. Infant health, height and weight were assessed periodically using standardized questionnaires [15]. 

For the analyses reported in the present study, HM samples were selected from 25 Dutch women participating in the PreventCD project. Inclusion criteria were that the participating mothers did not have CD and were consuming a normal, gluten-containing diet. Their infants (13 girls and 12 boys) had not developed CD at the age of 5–8 years. Infant weight and growth characteristics are represented in Table 1. 

### 2.2. Sample Collection

Participating women were instructed to collect 10–30 mL of HM at home manually or with an electric pump once a month, around the same day. After sampling, milk samples were frozen immediately at −20 °C overnight at the participant’s home, and were transferred the day after on ice to the hospital, where samples were stored at −80 °C until they were analyzed. The average milk sampling time in month 1 though month 6 of lactation was 24, 52, 81, 116, 148, and 178 days after delivery.

### 2.3. Amino Acid Analyses

A total of 135 HM samples were analyzed, comprising 25 series of monthly collected HM samples up to 6 months of lactation. Whereas most longitudinal sample series were complete some samples were missing, explaining the discrepancy in the number of milk samples analyzed in each month of lactation. Prior to AA and protein level analyses, the HM samples were thawed overnight at 4 °C and gently vortexed. Levels of FAAs (i.e., unbound AAs) and TAAs (i.e., free + bound AAs) were analyzed by the liquid chromatography method described in detail elsewhere [18], using an ultra-fast liquid chromatography system (Shimadzu, ‘s-Hertogenbosch, The Netherlands) which is equipped with a fluorimetric detector and an Acquity UPLC BEH C18 column (1.7 m, 100 × 2.1 mm) (Waters, Milford, MA, USA). This method does not allow the detection of proline and cysteine, yielding a total of 18 detectable FAAs, and taurine. The measurements of TAA levels required total protein hydrolysis using 6 M HCl, which enabled the detection of 15 TAAs and disabled the detection of tryptophan, cysteine, and proline. The acidic hydrolysis process also transformed asparagine into aspartate and glutamine into glutamate, disabling the detection of these TAAs individually. For AA peak identification, AA standards (Sigma, Zwijndrecht, The Netherlands) were used. The protein content per sample was estimated by multiplying the N-content by the conversion factor 6.25 [19]. The N-content in the HM samples was analyzed by the Dumas method, described elsewhere [20]. As opposed to TAA content, the total protein content also comprises the non-detectable AAs, as well as non-protein N (including a.o. taurine and nucleotides).

### 2.4. Statistical Analysis

Regression coefficient analysis was applied to analyze the effect of the lactation time on protein, TAA and FAA levels in HM. Briefly, for each subject, the protein, TAA and FAA levels were regressed against time through months 1–3 of lactation, months 4–6 of lactation or the entire 6-months study period. The rationale for using these time points for regression analysis was that existing effects of lactation time on the AA content in HM were clear in the first 3 months of lactation and less pronounced during months 4–6. Mean values of the slopes of the regression lines were tested against a hypothetical mean of 0 by means of a one sample *t*-test. Differences in cumulative levels of protein, TAAs and FAAs between milk for boys and milk for girls in month 1–3 of lactation were assessed by using a mixed model with subjects as random effect and the sampling time in days after delivery and gender as fixed effects. Missing data (*n* = 3 in months 1–3 of lactation) were considered missing at random. Statistical significance was defined as *p* < 0.05 and where applicable trends were indicated when *p* < 0.10. All data are reported as means ± SEM, unless indicated otherwise. Statistical analyses were performed using GraphPad Prism (version 7.03, GraphPad Software Inc., San Diego, CA, USA) and JMP (version 11.0.0, SAS Institute Inc., Cary, NC, USA).

### 2.5. Ethics

The PreventCD trial, registered as ISRCTN74582487, was approved by the medical ethics committee of the Leiden University Medical Center (LUMC) in the Netherlands on the 1st December 2006 and complied with the Good Clinical Practices (ICH-GCP) guidelines. The parents of the children provided their written informed consent for the study. The study was conducted according to the Declaration of Helsinki.

## 3. Results

Table 2 summarizes the FAA levels in HM during the first 6 months of lactation. As shown, glutamate was by far the most abundant FAA, increasing from 53.7% to 56.9% of the total FAA content. To prevent glutamate from masking and diluting the effects of the lactation time on levels of other FAAs, statistical calculations concerning the total FAA content were conducted with both inclusion and exclusion of free glutamate. Regression coefficient analysis revealed that the total FAA content with or without glutamate in HM significantly increased along the first 3 months of lactation, and remained stable thereafter, as shown in Table 2. Significant increases of individual FAAs were found for non-essential amino acids (NEAAs) aspartate, glutamate, glutamine, glycine and serine in the first 3 months of lactation. Most of the essential amino acids (EAAs) levels remained stable along the study period, only threonine and histidine levels showed a slight but significant increase and decrease in the first 3 months of lactation, respectively (Table 2).

In contrast to the sum of all FAAs, the protein content and the sum of all TAAs, as well as TAA levels of each of the individual AAs decreased in the first 3 months of lactation, followed by a gradual non-significant decline thereafter (Table 3). Consequently, the ratio of the sum of FAAs to the sum of TAAs significantly increased in the first 3 months of lactation (Table 4). With respect to individual AAs, the FAA/TAA ratio for Glx (glutamate + glutamine), Asx (asparagine + aspartate), serine, glycine, alanine, threonine and histidine increased significantly in month 1–3 of lactation, and the ratios remained stable thereafter. For all other AAs the increase of the FAA/TAA ratio was more gradual but also reached significance, or at least showed a trend, when regression coefficient analyses were performed over the entire study period (Table 4). The highest relative increases in the FAA/TAA ratio were observed for Glx, Asx, serine, and glycine in the first 3 months of lactation. 

**Figure 1:**
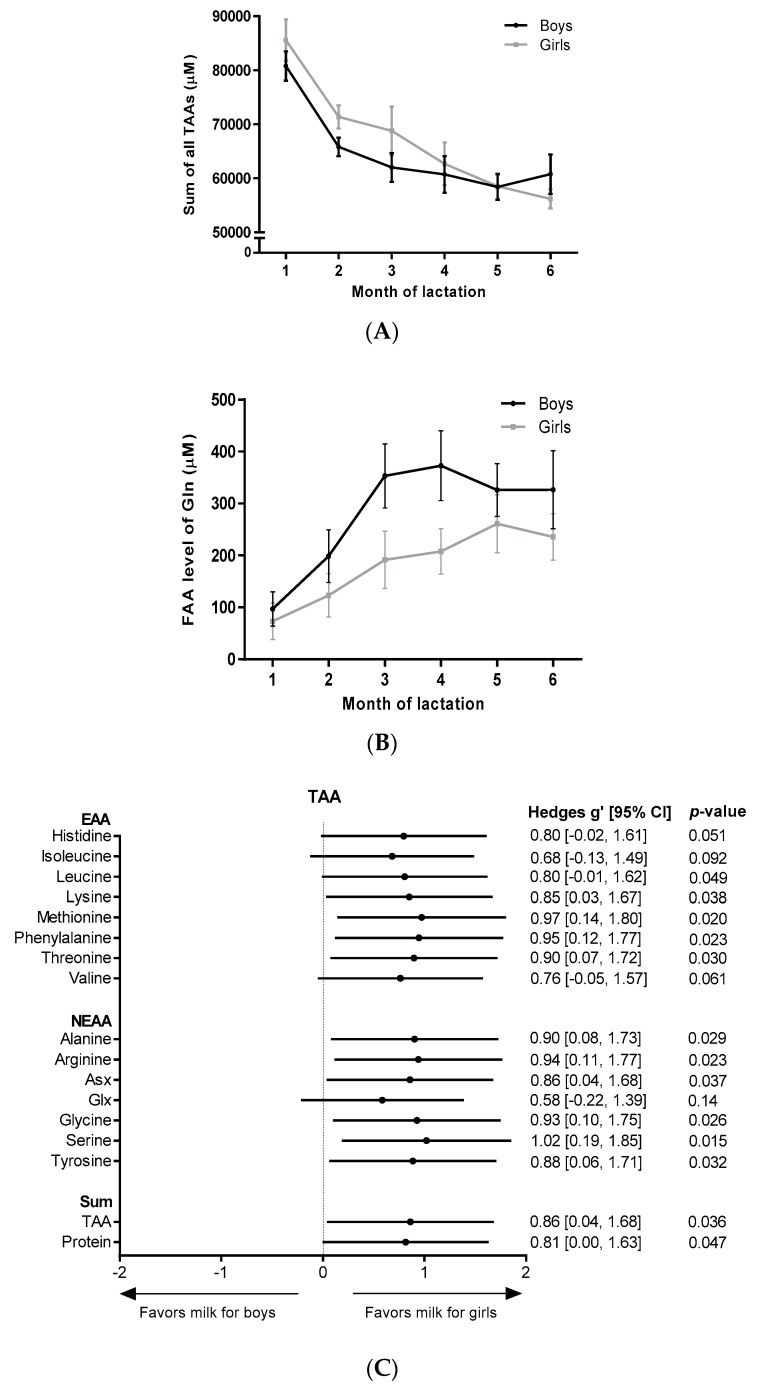

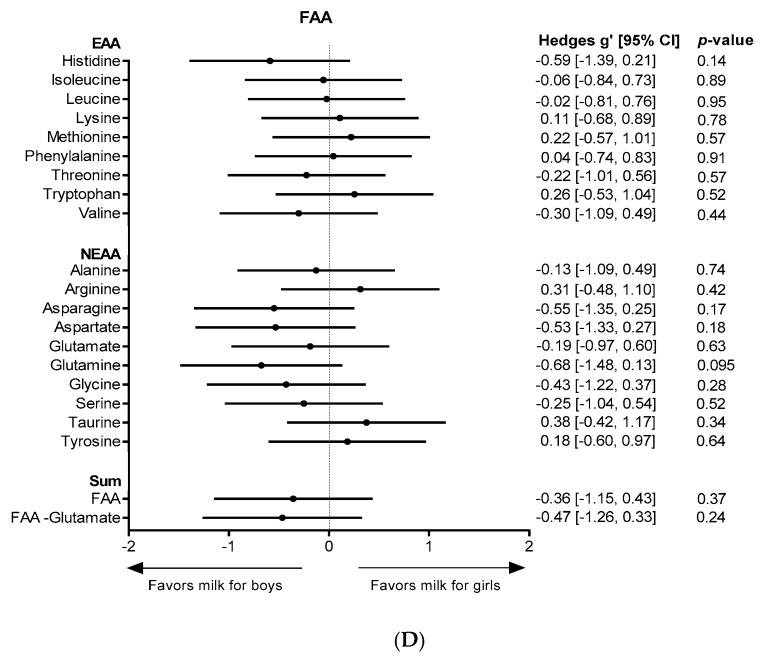
AA levels in milk for boys and milk for girls. Levels over the course of lactation are shown for (**A**) the sum of all TAAs, and (**B**) free glutamine. Forest plots indicating the effect sizes (Hedges’ g) of gender on the AA content in the first 3 months of lactation are shown for each individual TAA (**C**) and for each individual FAA (**D**). FAA: free amino acid; EAA: essential amino acid; NEAA: non-essential amino acid; Gln: glutamine, Asx: aspartate + asparagine; Glx: glutamate + glutamine.

Analyses of gender differences by fitting a mixed model (girls: *n* = 13, boys: *n* = 12) revealed a significantly higher sum of all TAAs (*p* = 0.0362) (Figure 1A) and total protein content (*p* = 0.0465) in milk for girls in the first 3 months of lactation. Milk for girls contained significantly higher TAA levels of each individual AA in the first 3 months of lactation, except for Glx (*p* = 0.1443), histidine (*p* = 0.0511), valine (*p* = 0.0612) and isoleucine (*p* = 0.0918) (Figure 1C). As represented in Figure 1C, effects sizes of gender were moderate (Hedges’ g = 0.5–0.8) for total Glx, histidine, valine and isoleucine and large (Hedges’ g ≥ 0.8) for all other measured TAAs. Gender had no significant effect on the FAA content in HM, however, a trend for higher levels in milk for boys was observed for free glutamine (*p* = 0.0948, Hedges’ g = 0.68) in the first 3 months of lactation (Figure 1B,D). No gender differences were found in months 4–6 of lactation.

## 4. Discussion

The composition of HM is considered optimal for supporting a healthy development of the newborn in the first months of life. In recent years FAAs in HM gained considerable attention and their changing composition along lactation has led researchers to discuss functional roles of FAAs in infant development. To contribute to this discussion, we studied the absolute and relative FAA levels in HM along the first 6 months of lactation and reported for the first time associations between FAA, TAA and protein levels in HM and infant gender.

The dynamics and levels of FAAs, TAAs and protein found in the present study were comparable to those reported in a systematic review by Zhang and colleagues [7]. We previously confirmed that the total protein and TAA content in HM decreased in the first 2 or 3 months of lactation with levels remaining relatively stable thereafter [21]. The decreasing TAA and protein levels have been shown to correlate with the infant requirements for growth and might prevent overfeeding of AAs, as milk volume intake per kg body weight increases with progressing lactation [22,23].

The present research showed that the FAA/TAA ratio almost doubled in the first 6 months of lactation, which might indicate an increasing importance of FAAs compared to TAAs for the developing infant. Consistent with other studies, the contribution of FAAs to TAAs in this study was 2.5% to 5%, depending on the time point during lactation [7,24,25]. Therefore, it is expected that the contribution of FAA to the infant’s nutritional requirements is low. Despite their low abundance, FAAs in HM contribute significantly to initial changes in the infant’s plasma FAA following a feed [24,26]. It is speculated that FAAs are more rapidly absorbed after ingestion than protein-derived AAs, appear more readily in the circulation and, thus, might reach peripheral organs and tissues faster [8,26,27]. Receptors recognizing specific FAAs are found on a wide variety of cells in multiple tissues, supporting the concept that specific FAAs in HM might be of physiological importance for the infant [9,10].

Whereas relative levels of almost all FAAs increased with progressing lactation, absolute levels of most FAAs remained stable along lactation. We found an increase of free aspartate, glutamate, glutamine, glycine, serine and threonine in months 1–3 of lactation, which are all polar amino acids in their free form. Other studies in a variety of ethnic groups found the same FAAs to increase along lactation, indicating a globally consistent FAA time course pattern in HM [7,9,25].

Our results showed that glutamate was by far the most abundant FAA and showed the highest absolute increase in concentration, followed by free glutamine. Similar observations have been reported by others [7,25]. The secretion of these FAAs in milk is an actively regulated process where branched-chain amino acids are catabolized in lactating mammary tissue to provide a.o. glutamate and glutamine, suggesting an important role of the two FAAs for the developing neonate [28]. Studies show that glutamine and glutamate support intestinal development, which is not yet completed at birth [29,30]. For instance, they are energy substrates for intestinal cells [31] and important for the generation of glutathione (glutamate) and epithelial tight junctions (glutamine), which are vital for the protection and maturation of the neonatal gut barrier [30,32,33,34]. It is postulated that glutamine and glutamate are implicated in the infant’s immune-development, as both AAs are indicated to modulate immune cell function and immune responses [29,35,36]. For example, at concentrations comparable to those in HM glutamine modulates T-cell proliferation, increases the production of T helper type 1 (TH1) cytokine IL-2 by T cells, and modulates differentiation of B-cells [35,37,38,39]. Glutamate has been demonstrated to modulate cytokine expression of dendritic cells and thereby drive the development of regulatory T cells and the inhibition of T helper type 2 (TH2) cells, and to stimulate the production of regulatory cytokine IL-10 by T-cells [33,36,40]. Many of these effects are TH1-skewing and/or TH2-inhibiting. Therefore, it can be speculated that free glutamine and glutamate could, next to other factors in HM, play a protective immune-modulating role against early life TH2-driven (food) allergy.

The present study reports several associations between infant gender and AA content in HM. Milk for girls was associated with higher levels of protein and TAAs, whereas milk for boys showed a trend towards higher levels of free glutamine when evaluated over the first 3 months of lactation. Interestingly, Thakkar et al. also demonstrated that the protein content in milk for girls was slightly higher than that in milk for boys in, at least, the first 60 days of lactation [41]. They, however, did not report significant differences, possibly due to them analyzing gender differences per time point measured, in contrast to our statistical method which includes the first 3 months of lactation simultaneously. As milk volume intake per kg of body weight is roughly similar for boys and girls, the observed differences might result in differences in AA intake. Larnkjær et al., recently showed that infant length at 4 months was positively associated with free glutamine levels in HM [42]. In compliance with this association, male infants in the present study tended to gain more length (*p* = 0.0556) during months 2–4 than female infants. Although Pearson correlation revealed a positive association of free glutamine in HM and infant length in the present study, this was not found to be significant.

The present study has several limitations. First, participating mothers gave birth to children genotypically at risk of CD (i.e., the presence of DQ2 and DQ8 haplotypes). However, we consider the present study to be representative for the general population as participating infants had not developed CD at 5–8 years of age, as the prevalence of DQ2 and DQ8 haplotypes in the general population is high (up to 30%), and as participating mothers did not have CD [43]. Another limitation is that milk sample collection was not strictly controlled as participants could sample from either breast and throughout the day. Sanchez et al. reported that levels of some AAs in HM might vary throughout the day, but the variations were only small [44]. In the present study, detailed information regarding maternal diet, other than that participating mothers consumed a free-living, gluten-containing diet, is lacking. Maternal diet might influence HM composition. However, the contribution of maternal diet to protein, FAA and TAA levels in HM is expected to be low, as those levels appear to be well preserved among mothers across different geographical locations consuming different diets [7,45,46]. This is reinforced by a study in China that reported the absence of a link between the AA concentration in maternal diet and HM [47]. A final limitation of the present research is that it lacks colostrum milk samples (0–5 days) and mainly analyzes mature HM (>15 days). The lack of colostrum milk samples is unfortunate, as effects of lactation stage on FAA levels in HM are shown to be best observed between colostrum and transitional milk. For instance, free glutamine levels are demonstrated to increase up to 7-fold between colostrum and transitional HM [7].

## 5. Conclusions

The present study demonstrated that levels of FAAs, TAAs, and protein in HM vary during lactation. For almost all individual AAs, the abundance of FAA increased relative to bound AA in the first 3 months of lactation. Significant increases of absolute levels of FAAs in the first 3 months of lactation were found for a.o. glutamine and glutamate, which are known to exhibit modulatory functions on intestinal health and the immune system, and are demonstrated to have T_H_1-skewing capabilities. Furthermore, although confirmation is necessary in view of the relatively small sample size, our study found differential effects of gender on TAAs and free glutamine levels in HM in the first 3 months of lactation. This provides opportunities to study HM AA composition in relation to (immune-related) diseases that occur differently between boys and girls in the first years of life.

## Figures and Tables

**Table 1:** Characteristics of the participating infants.

	Male (*n* = 12)	Female (*n* = 13)	Statistical Analysis of the Difference
Weight (g)			
Birth	3511.3 ± 144.3 (12)	3655.0 ± 104.1 (13)	0.4223
Δ 0–2 months	1996.7 ± 202.4 (12)	1406.5 ± 122.6 (10)	* 0.0279
Δ 2–4 months	1375.5 ± 187.5 (10)	1107.8 ± 78.8 (9)	0.2232
Δ 4–6 months	1033.6 ± 140.9 (7)	798.4 ± 97.6 (9)	0.1785
Δ 0–6 months	4156.7 ± 279.7 (9)	3392.8 ± 273.1 (9)	^o^ 0.0684
Length (cm)			
Birth	50.0 ± 0.7 (4)	50.8 ± 0.4 (9)	0.3327
Δ 0–2 months	6.6 ± 1.0 (4)	7.7 ± 0.6 (6)	0.3876
Δ 2–4 months	5.9 ± 0.5 (9)	4.4 ± 0.6 (8)	^o^ 0.0556
Δ 4–6 months	4.0 ± 0.6 (6)	3.6 ± 0.5 (8)	0.6312
Δ 0–6 months	17.2 ± 1.8 (4)	14.2 ± 0.9 (6)	0.1335

Values are given as mean ± SEM (*n*). Statistical differences in weight or height gain between male and female infants are indicated as follows: ^o^ *p* < 0.10, * *p* < 0.05.

**Table 2:** Free amino acid levels in human milk during months 1–6 of lactation.

FAA (µmol/L)	Month 1 (*n* = 23)	Month 2 (*n* = 25)	Month 3 (*n* = 24)	Month 4 (*n* = 18)	Month 5 (*n* = 23)	Month 6 (*n* = 22)	Statistical Analysis of the Changes
**EAA**							
Histidine	23.3 ± 1.4	21.8 ± 1.2	20.9 ± 1.1	19.2 ± 1.2	17.7 ± 0.7	18.5 ± 1.1	
Isoleucine	12.5 ± 2.2	12.4 ± 1.4	10.4 ± 1.1	12.3 ± 1.9	10.4 ± 0.8	14.4 ± 2.7	
Leucine	35.4 ± 5.1	36.0 ± 3.4	31.8 ± 2.2	40.8 ± 7.0	31.5 ± 2.3	40.1 ± 7.3	
Lysine	31.2 ± 4.1	28.5 ± 4.9	28.9 ± 3.1	28.7 ± 7.6	24.5 ± 2.0	26.9 ± 3.3	
Methionine	2.3 ± 0.5	3.0 ± 0.4	2.6 ± 0.2	3.2 ± 0.5	3.4 ± 0.3	3.9 ± 0.7	
Phenylalanine	18.8 ± 2.5	19.3 ± 1.6	17.4 ± 1.9	18.2 ± 1.8	15.5 ± 0.8	21.0 ± 3.9	
Threonine	66.6 ± 4.7	80.2 ± 6.0	75.4 ± 3.9	81.7 ± 6.4	85.1 ± 4.4	80.5 ± 5.2	a *, c *
Tryptophan	23.8 ± 1.3	23.3 ± 1.4	21.8 ± 1.5	22.3 ± 1.8	20.8 ± 1.2	24.1 ± 2.6	
Valine	54.2 ± 4.6	57.9 ± 3.5	53.3 ± 3.3	56.3 ± 3.9	45.6 ± 2.4	49.6 ± 3.7	
**NEAA**							
Alanine	251.5 ± 22.0	236.2 ± 13.6	241.7 ± 13.2	256.2 ± 15.8	245.3 ± 11.9	227.7 ± 16.4	
Arginine	16.4 ± 1.6	15.9 ± 2.5	13.4 ± 1.2	20.6 ± 5.9	15.3 ± 1.8	16.8 ± 2.0	
Asparagine	9.1 ± 1.9	9.5 ± 1.9	11.4 ± 2.4	12.9 ± 3.4	13.2 ± 2.3	10.1 ± 2.2	
Aspartate	39.8 ± 3.8	50.4 ± 4.0	61.8 ± 7.4	63.6 ± 5.9	74.4 ± 7.7	67.0± 7.0	a **, c **
Glutamate	1267.4 ± 84.3	1634.3 ± 62.1	1713.4 ± 54.6	1825.4 ± 66.1	1776.5 ± 60.4	1769.2 ± 70.9	a **, c **
Glutamine	84.1 ± 23.9	159.3 ± 32.9	265.6 ± 43.6	290.1 ± 43.8	293.4 ± 37.6	281.0 ± 43.3	a **, c **
Glycine	82.7 ± 7.9	94.7 ± 6.6	102.5 ± 6.4	104.1 ± 5.4	105.2 ± 5.2	111.5 ± 7.3	a **, c **
Serine	79.6 ± 6.7	97.4 ± 6.1	107.0 ± 7.1	107.3 ± 5.8	104.76 ± 6.4	100.0 ± 6.2	a *, c **
Taurine	244.1 ± 14.2	240.1 ± 16.6	250.0 ± 20.7	232.1 ± 19.6	218.0 ± 12.8	245.9 ± 26.9	
Tyrosine	16.8 ± 1.9	15.8 ± 1.5	13.8 ± 1.0	14.7 ± 1.7	14.0 ± 1.2	17.3 ± 1.8	
**SUM**							
EAA	284.7 ± 21.5	298.2 ± 20.0	268.9 ± 12.2	304.1 ± 29.8	269.7 ±11.3	295.7 ± 25.7	
NEAA	2075.0 ±129.1	2537.9±102.6	2767.2± 94.1	2906.3 ±107.4	2844.8 ±81.7	2829.8 ±118.6	a **, c **
FAA	2359.7 ±140.2	2836.1±111.5	3036.1 ±100.9	3210.4 ±117.8	3114.5 ± 88.1	3125.5 ±133.6	a **, c **
FAA without Glutamate	1092.3 ± 64.9	1201.8 ± 66.7	1322.7 ± 60.5	1385.1 ± 70.2	1338.0 ± 54.8	1356.2 ± 81.6	a **, c **

FAA: free amino acid; EAA: essential amino acid; NEAA: non-essential amino acid. Values are given as mean ± SEM. Significant changes (* *p* < 0.05, ** *p* < 0.01) of FAA levels over time as follows: a: Significant increase from months 1–6 of lactation; b: Significant decrease from month 1-6 of lactation; c: Significant increase from months 1–3 of lactation; d: Significant decrease from months 1–3 of lactation; e: Significant increase from months 4–6 of lactation; f: Significant decrease from months 4–6 of lactation.

**Table 3:** Total amino acid levels in human milk during months 1–6 of lactation.

TAA (µmol/L)	Month 1 (*n* = 23)	Month 2 (*n* = 25)	Month 3 (*n* = 24)	Month 4 (*n* = 18)	Month 5 (*n* = 23)	Month 6 (*n* = 22)	Statistical Analysis of the Changes
**EAA**							
Histidine	1872.9 ± 53.9	1530.9 ± 31.6	1462.9 ± 62.2	1368.1 ± 58.6	1278.0 ± 40.0	1259.7 ± 38.8	b **, d **
Isoleucine	5112.8 ±145.9	4235.2 ± 98.1	4071.5 ± 162.7	3825.6 ± 167.7	3544.6 ± 109.6	3339.8 ± 79.2	b **, d **
Leucine	9774.1 ±276.8	8094.0 ± 190.7	7748.7 ± 314.5	7299.3 ± 318.2	6813.3 ± 208.4	6667.9 ± 191.1	b **, d **
Lysine	6281.0 ±169.4	5154.2 ± 123.1	4902.8 ± 202.9	4578.6 ± 195.9	4281.1 ± 128.7	4259.6 ± 136.7	b **, d **
Methionine	1352.4 ±41.6	1068.5 ± 25.8	1028.2 ± 52.1	944.2 ± 43.2	879.8 ±3 1.0	832.6 ± 25.3	b **, d **
Phenylalanine	3021.2±91.9	2452.1 ± 61.3	2309.4 ± 107.9	2154.0 ± 93.3	2050.6 ± 62.3	2089.5 ± 89.3	b **, d **
Threonine	4800.2 ±142.3	3932.4 ± 94.4	3717.7 ± 166.6	3470.8 ± 144.1	3317.5 ± 96.5	3393.9 ± 146.2	b **, d **
Valine	5727.5 ±181.5	4691.9 ± 103.8	4546.9 ± 204.6	4289.8 ± 187.3	4072.3 ± 121.2	4041.1 ± 141.5	b **, d **
**NEAA**							
Alanine	5573.0 ± 173.6	4539.4 ±108.2	4280.9 ± 200.3	4015.8 ± 169.5	3891.3 ± 111.7	4068.7 ± 205.9	b **, d **
Arginine	2730.2 ± 104.5	2127.9 ± 64.6	2007.9 ± 117.4	1847.8 ± 87.7	1840.2 ± 64.1	1993.2 ± 140.6	b **, d **
Asx	9174.8 ± 278.4	7383.2 ± 165.1	6932.7 ± 315.1	6475.7 ±2 74.2	6171.5 ± 182.2	6189.0 ± 244.3	b **, d **
Glx	15,044.7 ± 397.7	13,123.7 ± 244.1	12,885.6 ± 433.4	12,331.7 ± 443.1	11,573.0 ± 294.3	11,274.9 ± 238.1	b **, d **
Glycine	4115.1 ± 130.9	3331.5 ± 95.2	3105.4 ± 161.5	2886.6 ± 121.0	2808.1 ± 87.1	3023.6 ± 180.1	b **, d **
Serine	5870.4 ± 180.6	4769.1 ± 111.4	4480.4 ± 202.7	4177.7 ± 172.4	3990.0 ± 118.2	4122.6 ± 200.5	b **, d **
Tyrosine	2848.9 ± 89.2	2285.1 ± 54.1	2204.0 ± 98.7	2044.1 ± 92.7	1949.8 ± 59.9	1929.7 ± 72.7	b **, d **
**SUM**							
TAA (mmol/L)	83.3 ± 2.4	68.7 ± 1.5	65.7 ±2 .8	61.7 ± 2.5	58.5 ± 1.7	58.5 ± 2.0	b **, d **
Protein (g/L)	14.4 ± 0.4	11.8 ± 0.2	11.3 ± 0.4	10.5 ± 0.4	10.0 ± 0.3	9.9 ± 0.3	b **, d **

TAA: total amino acid; EAA: essential amino acid; NEAA: non-essential amino acid; Asx: aspartate + asparagine; Glx: glutamate + glutamine. Values are given as mean ± SEM. Significant changes (** *p* < 0.01) of TAA levels over time as follows: a: Significant increase from months 1–6 of lactation; b: Significant decrease from months 1–6 of lactation; c: Significant increase from month 1–3 of lactation; d: Significant decrease from months 1–3 of lactation; e: Significant increase from months 4–6 of lactation; f: Significant decrease from months 4–6 of lactation.

**Table 4:** Percentage of free amino acids relative to total amino acids in human milk in months 1–6 of lactation.

FAA/TAA Ratio (%)	Month 1 (*n* = 22)	Month 2 (*n* = 25)	Month 3 (*n* = 24)	Month 4 (*n* = 18)	Month 5 (*n* = 23)	Month 6 (*n* = 22)	Statistical Analysis of the Changes
**EAA**							
Histidine	1.26 ± 0.083	1.45 ± 0.093	1.47 ± 0.095	1.41 ± 0.074	1.39 ± 0.058	1.50 ± 0.10	c *
Isoleucine	0.24 ± 0.040	0.30 ± 0.038	0.25 ± 0.022	0.33 ± 0.021	0.30 ± 0.025	0.36 ± 0.039	a ^o^
Leucine	0.36 ± 0.047	0.46 ± 0.048	0.42 ± 0.031	0.58 ± 0.10	0.47 ± 0.037	0.60 ± 0.10	a *
Lysine	0.50 ± 0.067	0.57 ± 0.10	0.45 ± 0.041	0.63 ± 0.16	0.58 ± 0.052	0.64 ± 0.082	a ^o^
Methionine	0.17 ± 0.038	0.28 ± 0.041	0.26 ± 0.026	0.44 ± 0.10	0.39 ± 0.038	0.40 ± 0.044	a **
Phenylalanine	0.61 ± 0.074	0.80 ± 0.071	0.74 ± 0.050	0.87 ± 0.084	0.76 ± 0.044	0.85 ± 0.066	a ^o^
Threonine	1.41 ± 0.11	2.09 ± 0.17	2.13 ± 0.16	2.38 ± 0.17	2.58 ± 0.16	2.50 ± 0.20	a **, c **
Valine	0.95 ± 0.076	1.27 ± 0.092	1.19 ± 0.075	1.35 ± 0.11	1.12 ± 0.057	1.20 ± 0.080	c *
**NEAA**							
Alanine	4.54 ± 0.41	5.28 ± 0.31	5.84 ± 0.35	6.39 ± 0.32	6.35 ± 0.037	5.81 ± 0.39	a *, c **
Arginine	0.60 ± 0.054	0.75 ± 0.12	0.68 ± 0.060	1.11 ± 0.31	0.86 ± 0.12	0.84 ± 0.072	a *
Asx	0.55 ± 0.064	0.84 ± 0.079	1.14 ± 0.17	1.21 ± 0.14	1.49 ± 0.17	1.33 ± 0.18	a **, c **
Glx	9.12 ± 0.77	13.81 ± 0.66	15.69 ± 0.76	17.50 ± 0.3	17.97 ± 0.69	18.33 ± 0.90	a **, c **
Glycine	2.04 ± 0.19	2.86 ± 0.18	3.37 ± 0.20	3.63 ± 0.16	3.77 ± 0.20	3.88 ± 0.27	a **, c **
Serine	1.39 ± 0.14	2.08 ± 0.15	2.50 ± 0.21	2.60 ± 0.12	2.59 ± 0.17	2.57 ± 0.21	a **, c **
Tyrosine	0.59 ± 0.064	0.71 ± 0.076	0.64 ± 0.047	0.72 ± 0.076	0.70 ± 0.059	0.85 ± 0.070	a *
**Total**							
FAA to TAA	2.56 ± 0.20	3.81 ± 0.19	4.36 ± 0.23	4.91 ± 0.26	4.97 ± 0.20	5.04 ± 0.29	a **, c **

FAA: free amino acid; TAA: total amino acid; EAA: essential amino acid; NEAA: non-essential amino acid; Asx: aspartate + asparagine; Glx: glutamate + glutamine. Values are given as mean ± SEM. Significant changes (^o^ *p* < 0.10, * *p* < 0.05, ** *p* < 0.01) of the percentage of FAA to TAA in time as follows: a: Significant increase from months 1–6 of lactation; b: Significant decrease from months 1–6 of lactation; c: Significant increase from months 1–3 of lactation; d: Significant decrease from months 1–3 of lactation; e: Significant increase from months 4–6 of lactation; f: Significant decrease from months 4–6 of lactation.
